# Avoidance, pacing, or persistence in multidisciplinary functional rehabilitation for chronic musculoskeletal pain: An observational study with cross-sectional and longitudinal analyses

**DOI:** 10.1371/journal.pone.0203329

**Published:** 2018-09-04

**Authors:** François Luthi, Philippe Vuistiner, Christine Favre, Roger Hilfiker, Bertrand Léger

**Affiliations:** 1 Department for Musculoskeletal Rehabilitation, Clinique Romande de Réadaptation suva, Sion, Switzerland; 2 Institute for Research in Rehabilitation, Clinique Romande de Réadaptation suva, Sion, Switzerland; 3 Department of Physical Medicine and Rehabilitation, Orthopedic Hospital, Lausanne University Hospital, Lausanne, Switzerland; 4 Unit of Psychosomatic Medicine, Clinique Romande de Réadaptation suva, Sion, Switzerland; 5 School of Health Science, University of Applied Sciences and Arts Western Switzerland Valais (HES-SO Valais-Wallis), Sion, Switzerland; University of Birmingham, UNITED KINGDOM

## Abstract

**Background:**

Three main activity patterns have been distinguished in describing chronic pain (avoidance, pacing and persistence). However, their influence on patient outcomes remains a question of debate. This observational study aimed to measure the associations between the avoidance, pacing, and persistence (labelled overdoing) scales of the Patterns of Activity Measure–Pain (POAM-P), self-reported outcomes (pain-interference, depression, functional ability), and observational outcomes (walking, lifting test, physical fitness).

**Methods:**

We conducted an observational study with cross-sectional and longitudinal analyses. The data were collected prospectively before and after treatment, which was a 5-week functional rehabilitation including vocational aspects. In addition to self-reported and observational outcomes, patients were asked if they thought they would be able to return to work at 6 months. Analyses were conducted with treatment effect sizes, correlations, and multiple regression models.

**Results:**

In this sample (891 patients), we found on average small to moderate improvements for pain-interference and observational outcomes (Cohen’s d: 0.37 to 0.64). According to the multivariable models, overdoing was associated with most of the beneficial psychosocial and observational outcomes (β -0.13 to 0.17; all p<0.01). Avoidance was related to negative psychosocial outcomes before treatment (β -0.09 to 0.17; all p<0.015). Pacing, which had moderate correlation with avoidance (r = 0.46), was not associated with most of the outcomes. The feeling that the goal of returning to work was attainable was associated with lower avoidance scores (adjusted OR 0.97; p = 0.024).

**Conclusions:**

The overdoing POAM-P scale probably measures a task-contingent persistence, which appears appropriate in the setting of this study. Persistent behavior was indeed related to small or moderate positive biopsychosocial outcomes, before and after treatment. Moreover feeling able to return to work was related to lower avoidance. Further studies should test the efficacy of motivational strategies that may promote functional task-contingent persistence and reduce avoidance of painful tasks.

## Introduction

Behavioral factors influence the development and perpetuation of musculoskeletal pain. Three main activity patterns have been described in patients with chronic pain: avoidance, pacing, and persistence[1]. Avoidance has been defined as a decrease in physical and daily activity, which occurs for several reasons such as fear of movement, fear of pain, or depressed mood[2]. Pacing is considered as a natural behavior to deal with chronic pain and activity[3], and is also a promoted strategy in numerous therapeutic programs[4, 5] as a balance between excessive inactivity and excessive activity. So far, there is no universally recognized definition of activity pacing. Nielson described it as a “regulation of activity level and/or rate in the service of an adaptive goal"[6]. It includes different strategies such as slowing down, alternating activity and pause, or dividing activity into smaller tasks. Persistence, also labeled as endurance, confronting, or overdoing, has received more attention over the past few years[7–9]. It has been defined as continuing activity despite pain, with high or more fluctuating levels of activity, and detrimental effects due to overuse[8].

Observational studies were conducted to study the association among these patterns and clinical outcomes. Avoidance has been the most studied and has consistently been associated with poor physical and psychological functioning and increase in pain[1, 10, 11]. The associations of pacing and persistence with physical and psychological outcomes are still controversial[1]. Commonly understood as a functional behavior, pacing has been recently challenged[8, 12]. McCracken found a positive relationship between pacing and disability[12]. Kindermans suggested that pacing may reflect some “hidden form” of avoidance[8], which is more pain-contingent and oriented on symptom reduction rather than on activity achievement. This assertion was supported by a recent meta-analysis suggesting some confusion between pacing and avoidance[13]. Persistence has been thought to be related to poor outcomes because of overuse and persistent soft tissue injuries[7, 14, 15]. Indeed excessive persistence or its combination with high avoidance level was associated with a higher level of anxiety and disability. However when persistence is associated with low avoidance or focused on task-contingence, it is related to higher functioning[8, 12].

To date, no theoretical models can explain all these different patterns. The Fear-Avoidance Model[16] is the most studied but it only investigates the avoidance pattern through fear mechanisms. Hasenbring, in her Avoidance-Endurance Model[14], has focused on the mechanisms of self-regulation of current activity goals, but only for avoidance and persistence patterns. She distinguished subgroups of patients (avoidant, distress endurance, eustress endurance) according to their strategies to face pain and activity. In her model, persistence is due to the suppression or minimization of pain-related thoughts. The Fear-Avoidance Model or the Avoidance-Endurance Model inherently associates a pattern with an outcome. Avoidance and distress-endurance patterns predict poor functioning, when the opposite is true for the eustress-endurance pattern without explicit reference to personal goals and to the context in which the behaviors occur. Moreover, these Models do not account for plausible combinations between each of these behaviors during the same period. For instance, it would be smart for a person with chronic knee pain to avoid running, but start walking or cycling to maintain his/her physical condition.

Recently, in a contextual-functional approach, Mc Cracken[17] proposed the model of psychological flexibility as a unifying framework for understanding activity patterns. The psychological flexibility represents the ability to consciously stay in touch with the present moment, without setting up unnecessary defences, and to put in place a behavior adapted to the service of a set personal goal[18]. One of the components of the model is the committed action which is defined as a value-oriented behavior pattern, persistent in that it incorporates setbacks or discomfort[19, 20]. The focus is set on goals and success instead of avoidance or persistence alone. Another component is the contextual Self. This conceptualization of the Self is centered on the capacity of perspective-taking which entails distancing oneself from one’s thoughts[18]. Recent studies have shown favorable associations between these components, social, and physical functioning[21]. The approach of Kindermans is based on the Self-discrepancies Model[22] and is also especially pertinent because pain often results in a loss of Self-confidence and in discrepancies in Self-images (the current Self vs the ideal Self). Behavioral activity strategies are seen as ways to solve these discrepancies[23] (persistence for staying the same person, avoidance for self-protection). Within these recent and more integrative perspectives, behavioral strategies are not functional or dysfunctional per se, their effects on disability and mood being more related to underlying personal goals and contextual factors[9]. Rehabilitation often offers different therapeutic contexts and objectives to improve the patient’s functioning (focusing on pain management, on functional restoration, or on vocational activity). These contexts may impact the patients’ main goals and consequently the relationship measured between behaviors and outcomes. For instance, a patient for whom being useful is positively valued may display persistence strategies in functional and vocational tasks despite pain, in an attempt to become as close as possible to the person he or she was before. Furthermore, if valued goals seem attainable, research suggests that people are prone to continue their efforts despite pain[24]. Recent experimental studies also have shown that a valued competing goal may attenuate avoidance behavior[25, 26].

We aimed to describe how the 3 activity patterns are related to pain-interference, depressive symptomatology, functional ability, and to measurements of walking, lifting test, and physical fitness, in a functional rehabilitation program including vocational aspects for patients with chronic pain. Activity patterns were assessed by using the Patterns of Activity Measure—Pain (POAM-P) developed by Cane[27] and recently validated in French[28]. In the original Cane study[27], avoidance and persistence (labelled overdoing) POAM-P scales were associated with negative psychosocial outcomes, whereas the pacing POAM-P scale was associated with positive outcomes. Interestingly and contrary to Cane’s results, Kindermans et al[8] found that the persistence POAM-P scale was predominantly related to a form of a functional “task-contingent persistence” negatively related to disability. Additionally the pacing scale of the POAM-P was positively related to disability and depressive symptomatology, which also disagreed with Cane’s results. However, no disagreement was found between the studies of Cane and Kindermans concerning the avoidance POAM-P scale. Based on these conflicting findings, other factors than the construction of the questionnaire may be considered to be infleuncial (for instance the clinical setting), and we cannot a priori deduce the direction of the future associations between persistence and pacing POAM-P scales and our judging criteria. In view of the above, we hypothesized that 1) persistence will be negatively related to pain-interference and depressive symptomatology and positively related to functional ability, physical activity, and improvements at discharge[22, 23]; and 2) avoidance will be positively related to pain-interference and depressive symptomatology and negatively related to functional ability, physical activities, and associated with lower improvements at discharge[10]. Due to the possible confusion between pacing and avoidance[13], we hypothesized that the associations between pacing and outcomes will be close to the associations observed with avoidance. Finally, the self-perceived ability of work resumption will be related to lower avoidance and higher persistence scores.

## Material and methods

### Design and setting

We conducted a single-center observational study with cross-sectional and longitudinal analyses in a Swiss rehabilitation hospital (Clinique Romande de Réadaptation, Switzerland). We measured pretreatment and posttreatment relationships between pain behaviors (avoidance, pacing, or persistence), and self-reported (questionnaires) and observational (performance-based measures) outcomes. The study was approved by the Local Medical Ethics Committee, Canton Wallis (CCVEM 034/12) and conducted in accordance with the Declaration of Helsinki. All patients read and signed an informed consent form. The STROBE checklist (S1 File) and the Data file (S2 File) are provided as supporting information.

### Participants

Participants were referred from all of the French-speaking counties of Switzerland, which includes urban and industrial city centers or more rural regions. The inclusion period was between October 2013 and June 2016. Patients of working age (18–65 years old) were sent to the functional multidisciplinary rehabilitation program by general practitioners, surgeons, or insurance medical advisors when they presented with chronic pain (for more than 3 months), functional impairments, and inability to return to work after orthopedic trauma injuries following work, traffic, sport, or leisure accidents[29]. Health and accident insurance are compulsory in Switzerland and patients are insured against occupational and non-occupational injuries. The insurer must pay for medical treatment as long as substantial improvement can be anticipated. The insured persons have legal rights for vocational measures, but they have to cooperate and to do everything possible to return to occupational activity[30]. The inclusion criteria was a musculoskeletal injury with more than 3 months of pain and disability. The exclusion criteria were traumatic brain injury at the time of the accident (Glasgow Coma Scale ≤ 8, to avoid possible interference between neuropsychological disorders and behaviors), spinal cord injury, incapable of judgment or under legal custody, and inability to complete the questionnaires in the local language (French). Indeed, many patients at our rehabilitation center are immigrant workers, 40% of them were not fluent in French[29] and were withdrawn from the study. However, patients who were non-native speakers but able to speak and understand French were included (38.4% of the study population). For patients who underwent multiple hospitalization periods, the first period only was considered for analysis.

### Therapeutic program

The aim of the therapeutic program was to manage pain, and to improve function, activity, and participation including return to work (usual or adapted), using a multidisciplinary biopsychosocial approach according to the recommended practice for patients with chronic pain [31]. This program is composed of physical components (physiotherapy and occupational therapy) with individual and group sessions including graded exercise (strength and endurance training, stretching, balance, walking, and adapted physical activities such as ball games, badminton etc) and psychological components. There was a mean of 4 psychological Cognitive-Behavioral Therapy (CBT) sessions, as well as social advice and vocational training. After determining the baseline physical capacity of the patient, therapies were determined by the therapists and then adjusted after a weekly multidisciplinary meeting. The length of stay was 4 to 5 weeks with at least 3 to 4 hours of daily therapy (excluding weekends). Patients had the opportunity to alternate activity and rest, according to their activity planning. Although the therapeutic program was not standardized for research purpose, its principles were graded activity with pacing strategies[5]. Physiotherapy and occupational therapy accounted for nearly 80% of all therapies.

### Measurements

#### Participants data

The following data were available from the medical chart: age, gender, marital status (yes vs no), education (≤ 9 years compulsory schooling vs > 9 years), professional qualification (yes vs no), severity of the injury (Abbreviated Injury Score (AIS)[32]: minor, moderate, or severe injury), pain localization (spinal: neck/back; upper limb; lower limb), co-morbidities assessed with the Cumulative Illness Rating Scale[33] (range, 0–56), and interval between injury and admission. As one of the main concerns of this functional rehabilitation program is to promote the return to work after injuries, all participants answered the following question at entry to estimate if they thought that this goal was attainable: “In 6 months, do you think you will have returned to work (yes/no)?”.

#### Questionnaires

**Activity patterns:** Avoidance, pacing, and persistence were assessed at entry by using the Patterns of Activity Measure—Pain (POAM-P) developed by Cane[27] and recently validated in French[28]. Unlike the avoidance and pacing patterns, persistence has received several labels: endurance, overactivity, persistence, and confrontation among others. It is labeled “overdoing” in the original Cane study. According to Cane’s choice, persistence will hence be labeled overdoing. POAM-P has good psychometric properties and identifies in clinical practice the 3 main activity patterns which all demonstrated excellent internal consistency (Cronbach’s alpha coefficients: 0.88, 0.89, and 0.85 for avoidance, pacing, and overdoing subscales respectively). The instructions are “People who have pain use different ways to do their daily activities. Think about how you usually do your daily activities”. Examples of item content are “I stop what I am doing when my pain starts to get worse” (avoidance), “I go back and forth between working and taking breaks when doing an activity” (pacing), and “When I am doing an activity I don’t stop until it is finished” (overdoing). The questionnaire has 30 items (10 for each pattern), coded from 0 to 4 points, and subjects have to choose to what extent the proposal applies to them. Each subscale is separately scored. The maximum score indicates a preferential but not exclusive behavioral pattern. In the absence of validated pattern classification methodology, each subscale will be handled with continuous scores according to previous research[27, 34, 35]. The POAM-P was only assessed at admission.

**Pain severity and Pain-interference:** To assess these 2 parameters we used the Brief Pain Inventory (BPI) questionnaire[36], divided into its 2 main subscales: pain severity scale (Cronbach’s alpha coefficient: 0.90), combining 4 different numeric rating scales (scale, 0–10) and the pain interference score combining seven different numeric rating scales (scale, 0–10). The interference scale (Cronbach’s alpha coefficient: 0.92), measures the interference of pain with general activity, mood, walking, normal work, relationships with others, sleep, and enjoyment of life. The BPI is considered as a pain-functioning measure[37]. According to the International Classification of Functioning[38], functioning refers to body function, activities and participation; similarly disability refers to impairment, activity limitation, and participation limitations. In others words, functioning is the positive manner to design disability.

**Pain-related fears:** We used the TAMPA scale of Kinesiophobia (TSK: range 17–68; Cronbach’s alpha coefficient: 0.71) to assess pain-related fears (fears of movement and re-injury), Higher scores indicate more pain-related-fears and were consistently related to higher pain and disability levels[39].

**Catastrophizing thoughts:** We used the Pain Catastrophizing Scale (PCS: range, 0–52; Cronbach’s alpha coefficient: 0.91)[40] to investigate catastrophic thinking (rumination, magnification, and helplessness) that is a risk factor for pain chronicity. Higher scores indicate more catastrophizing thoughts and are related to negative outcomes (for example, low return to work rate, more severe depressive symptoms, and more disability).

**Anxious and depressive symptomatology:** We used the Hospital Anxiety and Depression Scale (HADs: range, 0–21 for both scores; Cronbach’s alpha coefficients: 0.79 and 0.89 for anxiety and depression subscales respectively). Higher scores indicate greater anxious or depressive symptomatology, and a cut-off point of 8/21 is generally accepted for anxiety or depression symptoms[41].

**Functional Ability**: Functional ability represents a person's belief in his or her capability to perform a task/skill successfully[42]. Questionnaires assessing functional ability are therefore task-specific and mostly used in vocational literature. They do not measure social participation or others dimensions related to disability. For this purpose, we used the Spinal Function sort[43] (SFS: spinal and lower limb: 0–200, Cronbach’s alpha coefficient: 0.98) and the Hand Function Sort[44] (HFS: upper limb: 0–250, Cronbach’s alpha coefficient: 0.92). The SFS and HFS are pictorial questionnaires assessing specific daily life and working tasks/skills with good psychometric properties, and were developed to improve comprehension in patients with low literacy levels. For statistical purpose, both scores were normalized (0–200), and a score lower than 100 points indicates a poor functional ability, not able to adequately perform the usual tasks required in a sedentary activity, and a 200-point score, no limitation.

**Self-reported physical activity:** At entry, the leisure subscale (Cronbach’s alpha coefficient: 0.88) of the Baecke Physical Activity Questionnaire (BPAQ)[45] was used to assess the patient self-reported physical activity level before the admission. We didn’t consider sports and work subscales, as patients had legally stopped work for months, and cannot practice any sports due to their limitations.

All questionnaires used were the validated French versions[28, 46–52].

#### Observational measures

**Performance-based measures**: At entry and discharge, patients performed the 3 following observational measures: 1. The 6-minute walk test (6MWT)[53], a reliable and validated submaximal exercise test well designed for adults with pathology and chronic pain[54]. Participants were instructed to walk as fast and as far as they could for 6 minutes on a 120-meter walking track. The distance walked was recorded in meters; 2. The Progressive Isoinertial Lifting Evaluation (PILE)[55], a commonly used lifting test for patients with chronic pain. Participants were asked to lift a box 4 times in 20 seconds from the floor to the top of a 75-cm-high table, starting at 2.5 kg. After every completed cycle, the weight was increased by 2.5 kg for women and 5 kg for men. The test was stopped when the heart rate exceeded 85% of the maximal heart rate according to the age of the patient (ie, 220 minus age), if the subject was not able to maintain the lifting tempo, or if the maximum lifted weight exceeded 55% of the body mass; 3. The Steep Ramp Test (SRT) was performed to assess physical fitness[56]. After a warm-up of 2 minutes of unloaded cycling, the resistance was progressively increased (25 W/10s). Patients were instructed to cycle until exhaustion with a pedal frequency of 60–80 rotations per minute (rpm). The test was stopped when the pedal frequency dropped under 60 rpm. The SRT is an accurate test to estimate maximal aerobic capacity in untrained patients[57]. Performance-based measures have been added to the protocol since May 2014 (date of implementation in the rehabilitation program).

**Data collection and bias:** To minimize the measurement bias, questionnaires and outcomes at entry were collected before starting the therapeutic program. Records were collected electronically with a digital pen. The performance-based measures were done under the supervision of highly experienced physiotherapists, familiar with the tests through regular training and clear instructions. Questionnaires as well as physical tests were administered in the 2–3 days following entry, and the 2–3 days before discharge. Therapeutic teams were unaware of the pattern activity scores.

#### Outcomes

Self-reported evaluations (BPI-I, SFS/HFS, HAD-D), questions about the possibility of resuming work, and observational performance-based measures (6MWT, PILE, SRT) were assessed upon entry. Before discharge, we investigated the evolution of the scores of the self-reported and observational measures following the rehabilitation protocol.

### Statistical analysis

Descriptive statistics were expressed as mean and standard deviation for continuous variables, and as count and percentage for categorical ones. Influence of socio-demographic characteristics on POAM-P scores were assessed by means of *t* tests or ANOVA, as appropriate. Correlations between POAM-P scores and other continuous variables were also computed. Following Evans[58], the strength of correlations were described as follows: less than 0.20 is very weak, 0.20 to 0.39 is weak, 0.40 to 0.59 is moderate and 0.60 or greater is strong.

Associations between the self-reported and observational outcomes and the 3 subscales of the POAM-P were assessed by the mean of linear regression models. Associations between the possibility of resuming work and the 3 subscales of the POAM-P were assessed by the mean of logistic regression models. These associations were adjusted for age, gender, pain severity at admission, trauma localization, and education level, which could be potential confounding factors. At discharge, associations were also adjusted with the outcome baseline values as proposed by Senn [59], and with the length of hospitalization. Both unstandardized and standardized coefficients are provided. Standardized coefficients allow for a comparison of the associations between the different outcomes. Interactions between avoidance, pacing, and persistence were evaluated, but since none of them was significant, we did not consider them in the presented results. The absence of these interactions had already been observed in previous studies[27]. The available sample size of nearly 900 patients was comfortably sufficient to estimate the regression models given the low number of included covariates. The patients’ evolution between admission and discharge was assessed with mean differences and Cohen’s d. According to the literature, differences of 0.2, 0.5, and 0.8 were considered “small”, “medium”, and “large” effect sizes respectively[60].

It has been demonstrated [61] that patients with missing information have poorer treatment outcomes, for this reason, a complete-case analysis could result in biased results. Given the large number of explanatory variables collected, it can be assumed that the probability of missing values is not associated with unobserved data, the assumption of data being missing at random (MAR) is thus plausible, and multiple imputation should reduce the risk of bias[62]. Multiple imputation by chained equations (MICE)[63] was used to resolve missing values. Imputed datasets are obtained with the “mi impute chained” command of Stata 15.0. Using MICE is a practical approach for generating imputations, applying one imputation model for each variable with missing values. Continuous variables were imputed using linear regression models; logistic regression models were used for binary variables, and multinomial logistic regression for nominal variables. Normality was assumed for all continuous variables after visual inspection of the distributions. Each variable with missing values was regressed on all other variables, and missing values were replaced by simulated draws from the corresponding posterior predictive distribution. The number of imputed datasets is recommended to be at least equal to the percentage of incomplete cases[63]. In our data, 37.5% of patients had missing values, thus we used 40 imputations for the analyses. The 40 datasets were analyzed individually and the parameter estimates were combined into an overall estimate using Rubin’s rule [64]. Because performance-based measures were only introduced in May 2014, we did not impute these values for patients hospitalized before this date (N = 190). Analyses on subjective outcomes were performed on all 891 patients whereas those on performance-based outcomes were restricted to 701 subjects. Analyses were also performed on complete-case data only as a sensitivity analysis.

All statistical analyses were performed with Stata 15.0 (StataCorp, College Station, TX, USA). The limit for statistical significance was set as usual at p<0.05.

## Results

### Sample characteristics

**Fig 1:**
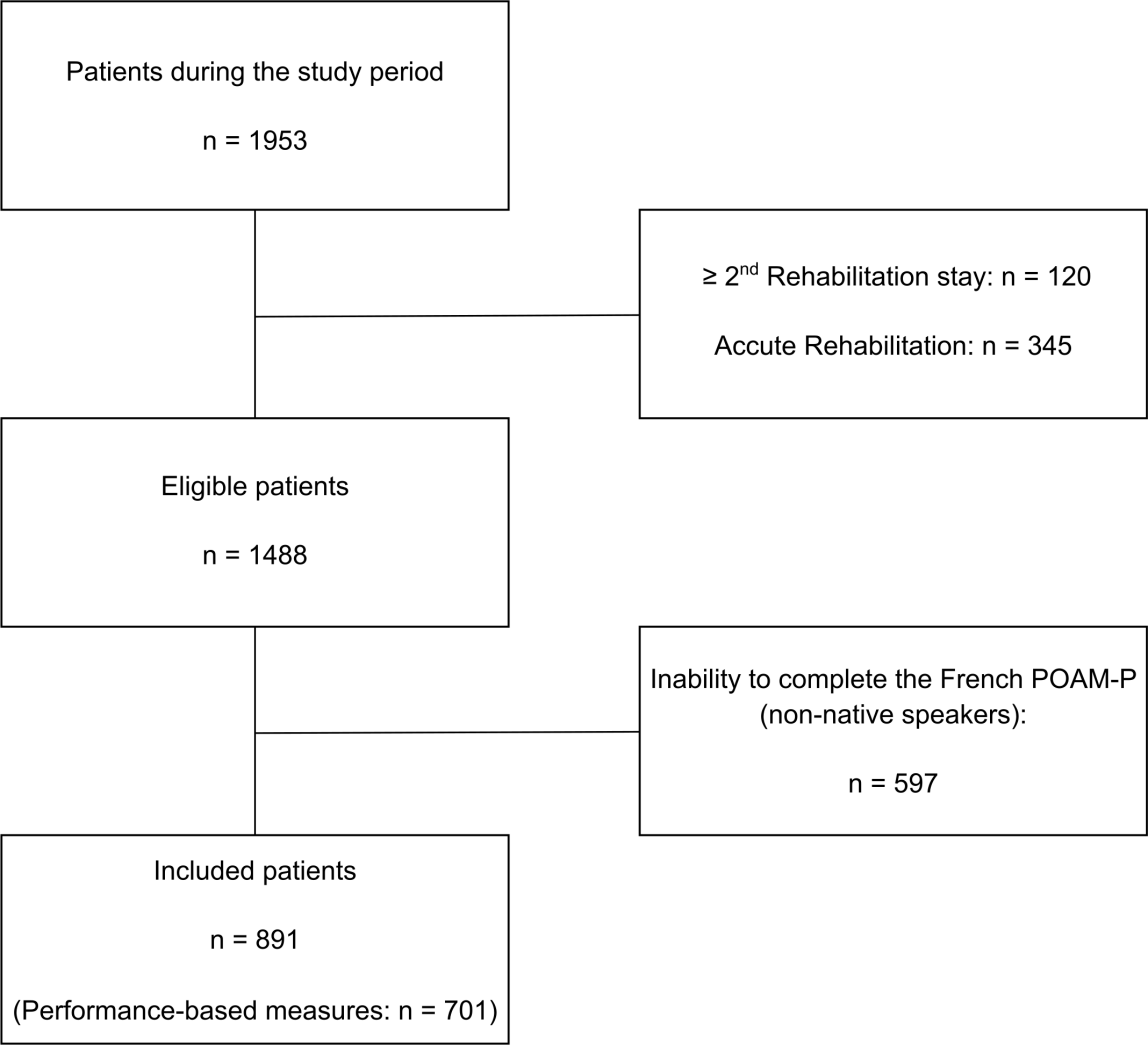
Flow chart of the study.

During the study period (Fig 1), 891 patients were included (mean age 43 years +/- 12; 18% female), among whom 701 completed the performance-based measures. Non-native speakers (but able to complete the questionnaires in French) represent 38.4% of the study population. The median of pain duration was 359 days (IQR: 233–678). Patients with spinal pain represented 16% of the total number of patients, with pain in the upper limb 37%, with pain in the lower limb 43%, and with multiple pain sites 4%. The vast majority were injured following minor or moderate orthopedic trauma (84%), that is, sprains and strains or simple uncomplicated fractures (AIS 1–2). Work-related injuries counted for 44%. The median duration of hospitalization was 29 days (IQR: 27–35). Other characteristics are shown in Table 1. All recruited patients completed the intervention.

**Table 1:** Summary statistics.

Type of variable	Variable	N	Possible values	mean (sd) or N (%)
**POAM-P scores**	**Avoidance**	891	**0–40**	28.3 (8.4)
** **	**Pacing**	891	**0–40**	25.6 (8.9)
** **	**Overdoing**	891	**0–40**	21.4 (8.6)
**Outcomes at admission**	**BPI-I**	875	**0–10**	4.58 (2.21)
** **	**HAD-D**	871	**0–21**	7.16 (4.27)
** **	**SFS/HFS**	861	**0–200**	114.84 (48.64)
** **	**6MWT**	668	** **	486.85 (132.17)
** **	**PILE**	579	** **	16.58 (8.69)
** **	**SRT**	565	** **	199.11 (94.08)
** **	**Ability of Return to Work**	771	**Yes**	506 (65.6)
** **			**No**	265 (34.4)
**Outcomes at discharge**	**BPI-I reduction**	846	** **	0.69 (1.86)
** **	**HAD-D reduction**	850	** **	0.30 (2.80)
** **	**SFS/HFS improvement**	835	** **	2.45 (33.01)
** **	**6MWT improvement**	626	** **	63.29 (93.02)
** **	**PILE improvement**	525	** **	3.16 (5.21)
** **	**SRT improvement**	527	** **	31.06 (56.05)
**Biological**	**Age**	887	** **	42.92 (11.87)
** **	**CIRS**	849	**0–56**	4.69 (2.97)
** **	**Gender**	891	**Female**	157 (17.6)
** **	** **		**Male**	734 (82.4)
** **	**Trauma location**	881	**Upper limb**	331 (37.6)
** **	** **		**Lower limb**	378 (42.9)
** **	** **		**Back**	140 (15.9)
** **	** **	** **	**Multiple trauma**	32 (3.6)
** **	**AIS**	860	**Minor**	295 (34.3)
** **	** **		**Moderate**	428 (49.8)
** **	** **	** **	**Serious**	137 (15.9)
**Psychological**	**TSK**	873	**17–68**	44.62 (7.89)
** **	**PCS**	871	**0–52**	22.52 (12.19)
** **	**HADs anxiety**	871	**0–21**	9.34 (4.31)
** **	**BPI severity**	875	**0–10**	4.45 (1.99)
** **	**BPAQ leisure**	450	**1–5**	3.15 (0.69)
**Social**	**Native language**	880	**French**	542 (61.6)
** **	** **		**Other**	338 (38.4)
** **	**Partnership**	880	**Yes**	538 (61.1)
** **	** **		**No**	342 (38.9)
** **	**Education**	878	**> 9 years**	493 (56.2)
** **	** **		**≤ 9 years**	385 (43.9)
** **	**Full time work**	875	**Yes**	715 (81.7)
** **	** **		**No**	160 (18.3)
** **	**Work-related injury**	866	**Yes**	382 (44.1)
** **			**No**	484 (55.9)

Possible values indicate the range for continuous variables, and the categories used for binary variables. Descriptive statistics indicate the mean value and the standard-deviation in brackets for continuous variables, and the absolute number and relative number in brackets for each category for binary variables. BPI-I = Brief Pain Inventory—Interference; HAD-D = Hospital Anxiety and Depression Scale—Depression; SFS/HFS = Spinal Function Sort/Hand Function Sort; 6MWT = 6-minute walk test; PILE = Progressive Isoinertial Lifting Evaluation; SRT = Steep Ramp Test; CIRS = Cumulative Illness Rating Scale; AIS = Abbreviated Injury Scale; TSK = Tampa Scale of Kinesiophobia; PCS = Pain Catastrophizing Scale; HADs = Hospital Anxiety and Depression Scale; BPAQ = Baecke Physical Activity Questionnaire.

Higher avoidance scores were found in cases of minor injuries (AIS 1), work-related injuries, a lower level of education, non-native speakers, and male patients. Higher scores of pacing were found in non-native speakers, a lower level of education, and stable partnership, whereas higher scores of overdoing were found in female patients, serious injuries (AIS ≥ 3), native speakers, non-occupational injury, and patients with more than 9 years of compulsory schooling. Lower avoidance scores were related to the self-perceived ability to return to work. All these differences were statistically significant (Table 2). Effect sizes (eta-squared) were small to medium[65].

**Table 2:** Mean scores on the three POAM-P scales according to sociodemographic variables.

			Avoidance			Pacing			Overdoing		
Variable	N	Possible values	Mean	Eta-Squared	p-value	Mean	Eta-Squared	p-value	Mean	Eta-Squared	p-value
**Gender**	891	**Female**	26.39	0.011	0.002	25.04	<0.001	0.388	22.88	0.007	0.015
** **		**Male**	28.74			25.71			21.04		
**Trauma location**	881	**Upper limb**	28.61	0.007	0.110	25.89	0.006	0.173	20.42	0.009	0.052
** **		**Lower limb**	28.33			25.12			21.84		
** **		**Back**	28.52			26.66			22.16		
** **	** **	**Multiple trauma**	24.81			23.63			23.13		
**AIS**	860	**Minor**	29.24	0.013	0.003	25.48	0.001	0.661	20.37	0.008	0.029
** **		**Moderate**	28.43			25.80			21.73		
** **	** **	**Serious**	26.31			25.03			22.50		
**Native language**	880	**French**	26.63	0.065	<0.001	24.57	0.022	<0.001	22.44	0.023	<0.001
** **		**Other**	31.05			27.25			19.77		
**Partnership**	880	**Yes**	28.60	0.001	0.265	26.19	0.007	0.012	21.47	<0.001	0.796
** **		**No**	27.95			24.64			21.31		
**Education**	878	**> 9 years**	26.55	0.058	<0.001	24.45	0.022	<0.001	22.22	0.012	0.001
** **		**≤ 9 years**	30.63			27.08			20.32		
**Full time work**	875	**Yes**	28.57	0.002	0.194	25.48	0.002	0.233	21.30	0.001	0.397
** **		**No**	27.61			26.40			21.94		
**Work-related injury**	866	**Yes**	29.08	0.006	0.023	25.94	0.001	0.340	20.44	0.010	0.003
		**No**	27.78			25.36			22.20		
**Ability of Return to Work**	771	**Yes**	27.77	0.017	<0.001	25.32	0.001	0.403	21.88	0.003	0.138
		**No**	30.05			25.88			20.92		

Student t-tests or ANOVA are used to compare the groups. AIS = Abbreviated Injury Scale; Effect size (Eta-Squared): 0.01 = small; 0.06 = medium; 0.14 = large)

All correlations are presented in Table 3. The correlations should be interpreted as follows: less than 0.20 is very weak, 0.20 to 0.39 is weak, 0.40 to 0.59 is moderate and 0.60 or greater is strong [58]. Most of the correlations were weak and only correlations that are at least moderate are listed here. A moderate positive correlation was found between the avoidance and the pacing scores of the POAM-P (r = 0.46). Moderate positive correlations were also found between the avoidance score and the scores for kinesiophobia (r = 0.52), and catastrophizing (r = 0.47).

**Table 3:** Correlations between the three POAM-P scales and biopsychosocial factors.

			Avoidance		Pacing		Overdoing	
Variable	N	Possible values	Correlation	p-value	Correlation	p-value	Correlation	p-value
**Avoidance**	891	**0–40**	1.00		0.46	<0.001	-0.35	<0.001
**Pacing**	891	**0–40**	0.46	<0.001	1.00		-0.15	<0.001
**Overdoing**	891	**0–40**	-0.35	<0.001	-0.15	<0.001	1.00	
**BPI-I**	875	**0–10**	0.33	<0.001	0.09	0.010	-0.20	<0.001
**HAD-D**	871	**0–21**	0.28	<0.001	0.03	0.355	-0.25	<0.001
**SFS/HFS**	861	**0–200**	-0.27	<0.001	-0.12	<0.001	0.31	<0.001
**6MWT**	668	** **	-0.15	<0.001	-0.15	<0.001	0.14	<0.001
**PILE**	579	** **	-0.13	0.001	-0.10	0.018	0.10	0.012
**SRT**	565	** **	-0.18	<0.001	-0.20	<0.001	0.16	<0.001
**BPI-I reduction**	846	** **	0.02	0.640	-0.01	0.744	0.10	0.003
**HAD-D reduction**	850	** **	-0.07	0.032	-0.03	0.379	0.09	0.012
**SFS/HFS improvement**	835	** **	-0.03	0.374	0.01	0.881	0.05	0.118
**6MWT improvement**	626	** **	-0.05	0.226	0.04	0.265	0.11	0.009
**PILE improvement**	525	** **	0.03	0.470	0.04	0.359	0.06	0.198
**SRT improvement**	527	** **	-0.02	0.572	0.04	0.348	0.03	0.452
**Age**	887	** **	0.05	0.110	0.22	<0.001	-0.07	0.028
**CIRS**	849	**0–56**	0.03	0.367	0.05	0.169	-0.03	0.314
**TSK**	873	**17–68**	0.52	<0.001	0.20	<0.001	-0.29	<0.001
**PCS**	871	**0–52**	0.47	<0.001	0.14	<0.001	-0.26	<0.001
**HADs anxiety**	871	**0–21**	0.25	<0.001	0.00	0.993	-0.10	0.003
**BPI severity**	875	**0–10**	0.36	<0.001	0.12	<0.001	-0.24	<0.001
**BPI highest pain**	872	**0–10**	0.30	<0.001	0.07	0.035	-0.19	<0.001
**BPAQ leisure**	436	**1–5**	-0.04	0.400	-0.02	0.620	0.11	0.016

BPI-I = Brief Pain Inventory—Interference; HAD-D = Hospital Anxiety and Depression Scale—Depression; SFS/HFS = Spinal Function Sort/Hand Function Sort; 6MWT = 6-minute walk test; PILE = Progressive Isoinertial Lifting Evaluation; SRT = Steep Ramp Test; CIRS = Cumulative Illness Rating Scale; TSK = Tampa Scale of Kinesiophobia; PCS = Pain Catastrophizing Scale; HADs = Hospital Anxiety and Depression Scale; BPAQ = Baecke Physical Activity Questionnaire

### Relationships between patterns, self-reported questionnaires, and observational measures at entry

According to the multivariable regression models (Table 4), a higher score of the avoidance scale was related to higher pain-interference score (β coefficient 0.16), more depressive symptoms (β coefficient 0.17), and lower functional ability (β coefficient -0.09). No association was found between the avoidance score and the performance-based measures. A higher pacing score was not only related to less depressive symptoms (β coefficient 0.10) but also to lower performance in the 6MWT (β coefficient 0.10). A higher overdoing score was related to less depressive symptoms, higher functional better performances during all performance-based measures (β coefficient between– 0.13 and 0.17).

**Table 4:** Associations between the outcomes and the three POAM-P scales.

	Avoidance			Pacing			Overdoing		
Outcomes at admission*	**beta**	**coef**	**p-value**	**beta**	**coef**	**p-value**	**beta**	**coef**	**p-value**
**BPI-I**	0.16	0.04	<0.001	-0.05	-0.01	0.073	-0.02	-0.01	0.459
**HAD-D**	0.17	0.09	<0.001	-0.10	-0.05	0.005	-0.13	-0.07	<0.001
**SFS/HFS**	-0.09	-0.51	0.015	0.01	0.06	0.743	0.17	0.99	<0.001
**6MWT**	0.03	0.40	0.547	-0.10	-1.50	0.008	0.10	1.54	0.005
**PILE**	-0.08	-0.08	0.084	-0.01	-0.01	0.835	0.13	0.14	0.001
**SRT**	-0.07	-0.75	0.144	-0.08	-0.84	0.058	0.15	1.68	<0.001
** **		**OR**	**p-value**		**OR**	**p-value**		**OR**	**p-value**
**Ability of Return to Work**		0.97	0.024		1.01	0.215		1.00	0.864
Outcomes at discharge**	**beta**	**coef**	**p-value**	**beta**	**coef**	**p-value**	**beta**	**coef**	**p-value**
**BPI-I reduction**	-0.03	-0.01	0.512	0.00	0.00	0.901	0.12	0.03	<0.001
**HAD-D reduction**	-0.09	-0.03	0.042	0.04	0.01	0.325	0.09	0.03	0.011
**SFS/HFS improvement**	-0.05	-0.21	0.184	0.04	0.15	0.291	0.12	0.46	0.001
**6MWT improvement**	-0.05	-0.53	0.290	0.03	0.30	0.485	0.12	1.25	0.003
**PILE improvement**	0.02	0.01	0.689	0.02	0.01	0.675	0.11	0.07	0.016
**SRT improvement**	-0.02	-0.14	0.689	0.02	0.14	0.642	0.06	0.41	0.163

* adjusted for age, gender, pain severity, education level, and trauma location

** adjusted for outcome at admission, age, gender, pain severity, education level, trauma location, and length of hospitalization

Regression models for outcomes at admission are adjusted for age, gender, pain severity and trauma location. Models on outcomes at discharge are further adjusted for the value at admission, and the length of hospitalization. Linear regression models were performed on both standardized (beta) and unstandardized (coef) outcomes. Logistic regression was used for Ability of Return to Work (Odds Ratio). BPI = Brief Pain Inventory; HAD-D = Hospital Anxiety and Depression Scale—Depression; SFS/HFS = Spinal Function Sort/Hand Function Sort; 6MWT = 6-minute walk test; PILE = Progressive Isoinertial Lifting Evaluation; SRT = Steep Ramp Test.

### Relationships between patterns and the perceived ability of returning to work at 6 months

The avoidance score was significantly lower if the self-perceived ability of work resumption was viewed as attainable. For each 1 point difference in the avoidance score (0–40 points) the adjusted Odds Ratio was 0.97 (p = 0.024). No association was found with the pacing or overdoing scores (Table 4).

### Treatment effect sizes and relationships between patterns and outcomes at discharge

Considering the total sample (Table 5), differences in pain-interference and observational outcomes were all statistically significant, which meant at least small to moderate improvements between entry and discharge (Cohen’s d from 0.37 to 0.64). However, depressive symptoms and functional ability did not improve according to the Cohen’s d[60] (0.08 and 0.10, respectively).

**Table 5:** Mean difference between admission and discharge, both unstandardized (delta) and standardized (Cohen’s d).

	delta	Cohen's d	p-value*
**BPI-I**	0.69	0.37	<0.001
**HAD-D**	0.29	0.10	0.003
**SFS/HFS**	2.70	0.08	0.018
**6MWT**	63.16	0.64	<0.001
**PILE**	2.98	0.51	<0.001
**SRT**	29.51	0.44	<0.001

*t-test

BPI-I = Brief Pain Inventory—Interference; HAD-D = Hospital Anxiety and Depression Scale—Depression; SFS/HFS = Spinal Function Sort/Hand Function Sort; 6MWT = 6-minute walk test; PILE = Progressive Isoinertial Lifting Evaluation; SRT = Steep Ramp Test.

Regarding the relationships between activity patterns and improvements during the rehabilitation stay, overdoing was associated with pain-interference and depressive symptom reductions, functional ability improvement, as well as better walking and lifting performances (β coefficient between 0.10 to 0.13), the higher the overdoing score the higher the improvement (Table 4). No other significant relationship was found with the avoidance and pacing scores.

### Sensitivity analysis

The same analyses as presented above were computed on complete-case data. No noteworthy differences were observed compared to multiple imputation analyses in multivariable regression models (S1 Table). When measuring evolution between admission and discharge, the same conclusions would be drawn (S2 Table).

## Discussion

### Key findings

This study included a large cohort of patients with musculoskeletal chronic pain undergoing functional and vocational rehabilitation, and for the total sample we found on average small to moderate improvements for the different outcomes. Our results on treatment effects are similar to previous studies [66]. Regarding the possible influence of activity patterns, our results are only partially in accordance with our hypotheses.

As expected, before rehabilitation, overdoing was positively related to functional ability and physical performances and negatively related to depressive symptomatology. However we failed to demonstrate a relationship with pain-interference. At discharge, overdoing was also positively related to improvements with all self-reported and observational measures, except for physical fitness (SRT).

Regarding avoidance and in accordance with our hypotheses a positive relationship was observed with pain-interference and depressive symptomatology before rehabilitation, whereas functional ability was negatively related. Interestingly no relationship was found with physical performance. The small association between avoidance and pain-interference shows that they are 2 distinct contructs. At discharge, there were no self-reported or observational measures significantly related to the avoidance pattern. Our assumptions concerning the ability to resume work were partially confirmed here. Indeed the avoidance score was negatively related to the perceived ability to resume work, while no relationship was found with the 2 other patterns.

Few relationships were found for the pacing pattern. Before rehabilitation, pacing was negatively related to depressive symptomatology and lower walking performance. At discharge, pacing was not associated with any changes from baseline.

Others significant results, pointed towards moderate correlations between the avoidance and pacing POAM-P scores. A lower level of education was also significantly associated with higher avoidance and pacing scores, as well as lower overdoing scores. We found similar associations with the non-native speakers. However, the positive significant association found between minor injuries and avoidance score seems to be the result of probable selection bias; patients who combine less severe injuries with higher avoidance are most likely to be referred to rehabilitation[67].

### Strengths

The strength of this study relies on its large sample of patients and measurement of several self-reported and observational outcomes. Furthermore, we collected longitudinal data for the association of baseline behavior with the changes in outcomes. Our study enlarges the existing body of literature, which mainly focuses on patients with nonspecific pain syndrome with many years of pain history. Most of our patients were in a professional reintegration program with a 1-year average pain duration, and the functional and vocational setting of our project may shed a new light on the possible relationships between patients’ goals and activity patterns. Self-regulation and motivational theories, indeed, postulate that behaviors should be studied for their relationship with identity and valued goals[9]. In the context of functional and vocational rehabilitation, a person in pain who valued returning as a “good worker” after an injury (in others words to become as close as possible to the same person he or she was before), may display more efforts despite pain which seems in accordance with our results. In particular, if a valued goal seems attainable, previous research suggests that this may contribute to continuing efforts[24] and may therefore reduce avoidance behavior, which also seems consistent with our results.

### Interpretation

Results on the overdoing behavior may simply due to the operationalization of this behavior in the POAM-P questionnaire. Kindermans has conducted a factor analysis on 4 questionnaires assessing activity patterns, including the POAM-P[8]. She found a 3-factor structure for persistence behavior (considered as a synonym for overdoing): task-contingent persistence (finishing tasks or activities despite pain), excessive persistence (doing too much and experiencing rebound effects), and pain-contingent persistence (the level of pain being the determinant of the performed behavior). In her study, task-contingent persistence was negatively related to disability, whereas excessive persistence was positively related to disability and depressive symptomatology, and no relationship was found for pain-contingent persistence. In the POAM-P overdoing scale, 6/10 items were classified within the task-contingent persistence and the last 4 within the pain-contingent. The choice of the POAM-P would therefore explain our findings. However, this is not in line with Cane’s results, who developed the POAM-P[27]. He found that overdoing was positively related to disability and depression. These conflicting results means that other underlying factors probably play a crucial role. The setting of the 3 studies (Kindermans, Cane, and our study) was completely different: Cane’s study took place in a pain-management program with patients in pain for years, the study by Kindermans recruited participants who were not seeking care, through advertisements in local newspapers, whereas our patients were included in a functional and vocational rehabilitation program. From our point of view, this reinforces the idea that behavior, even the task-contingent persistence, may not be functional or dysfunctional per se. The relationships between behaviors and outcomes should be interpreted with caution, taking into account the role of contextual factors (ie, personal and environmental factors). It should also be emphasized that the way to designate this pattern can be a source of confusion. In the POAM-P, the overdoing subscale reflects the way the developer wanted to assess excessive persistence, whereas our results, in line with those of Kindermans, suggest that this questionnaire primarily measures a task-contingent persistence. As there are different ways of persisting in chronic pain, it could be less confusing to adopt a descriptive approach as proposed by Kindermans (task-contingent persistence, pain-contingent persistence, or excessive persistence)[8].

Regarding personal factors, models like that of Self-Discrepancy, seemed pertinent if considering the function of activity behaviors as an attempt to restore or to protect personal identity. Self-discrepancies with the fear- self (the self one fears to become) was positively related to avoidance behavior. However, the expected positive relationship between persistence and the ideal or ought self was not fully confirmed [19, 20]. A positive relationship with persistence was only found for patients who felt close or distant to the ideal self. Research on goal pursuit and motivation seems promising. Affleck showed that goals more highly valued were pursued with more effort in women with fibromyalgia[21]. In healthy volunteers, the pursuit of a valued goal, even in the presence of competing goals, was related to lower avoidance[22, 23], but to date this is without an attested relationship with persistence. The psychological flexibility model emphasises the goal-directed behavior and the flexibility to persist or to stop the activity according to the context and the capacity of the Self to decenter from its emotional experience[17]. Dedicated interventions to improve committed action (which includes a kind of activity persistence) and “Self-as-Context”, have shown significant benefits in functioning, and were also useful to reduce fears and avoidance[21, 68]. Both models of self-discrepancy and psychological flexibility broaden the understanding of activity patterns by including the role of the Self and personal values. They may emphasize complementary aspects of functioning. The Self-discrepancy model focuses more on content (how far the current Self is from the ideal or the Self one ought to be, and what to do, or how, to reduce it), the flexibility model focuses more on process (distancing from the content of the personal experience about the Self and committing in valued-action). Including the context and the Self might also suggest considering activity patterns as different behavioral strategies that are useful depending on the context and adaptive as far as they are flexible.

The possible role of education remains largely unexplored. McCracken found a significant lower education level in the avoiders, compared to the medium cyclers (patients with high persistence level and medium pacing and avoidance)[12]. We also found that a low education level was greatly related to higher avoidance and pacing scores, but lower overdoing scores. Cultural backgrounds (non-native speakers) were also highly related to similar results. Interestingly, a study on causal attribution differences between immigrant and non-immigrant populations with back pain found that immigrants were more prone to consider physical activity as an attributed cause of back pain[69], which could encourage avoidance behavior.

Our other findings on the avoidant pattern are in accordance with a relevant body of literature, patients adopting this behavior showing consistently poorer outcomes[10]. However, adverse relationships were mostly found with self-reported outcomes, which suggests that self-reported and observational tools assess different components of functioning[70], self-perceptions and performances. Self-reported measures are often considered as less burdensome and more cost-effective. Our results suggest that the use of one in place of the other is not without consequences. Without triangulation of results obtained from both approaches, researchers and clinicians may miss pertinent information. Huijnen also observed higher levels of self-reported disability in avoidance but without differences in observational daily activity levels[71]. These results highlight the interest in mixing self-reported with observational outcomes. Awareness of such differences may also be an important therapeutic target/tool to challenge adverse beliefs in rehabilitation and pain-management programs.

In our sample, pacing was not associated with the baseline or post-treatment outcomes, with the exception of a negative relationship with depressive symptoms and weaker walking performance at baseline. In his original study[27], Cane found that pacing scores were related to lower levels of depression and anxiety and greater perceived pain control. No association was found with disability. Pacing was challenged in other studies[8, 12, 72]. McCracken found a positive relationship between pacing and disability[12]. Murphy also found that an increased use of pacing was associated with lower physical activity[72]. Additionally, dysfunctional pacing reflecting some form of “hidden avoidance” was also suggested[8]. A recent meta-analysis[13] also found a small positive correlation between these activity patterns (r = 0.29, with correlation between low (r = 0.25)[27] to moderate (r = 0.54)[8]). We found a similar moderate positive relationship between avoidance and pacing (r = 0.46). These results perhaps suggest some confusion between pacing and avoidance, and may be due to unclear definitions of pacing and because existing questionnaires do not adequately assess pacing as a non-avoidant strategy aimed to reduce disability[13, 73]. There might also be 2 other explanations: 1) different goals or intents of pacing, and 2) realistic interpretation of his or her own situation. Regarding goals or intents of pacing, it has been proposed[73] that this parameter should be included in future tools. Recent efforts have been made to take into account these criticisms. Esteve developed the “Activity Patterns Scale” including 3 pacing subscales differentiated by the goal of the behavior[74]. In this study, pacing for increasing activity levels or conserving energy for valued activities was positively associated with daily functioning, whereas pacing for pain reduction was not. Regarding the second explanation, pacing scales should also be improved to better distinguish patients with a realistic interpretation of their own situation and those who pace because of other reasons, such as an unfounded fear of re-injury, which may be another source of confusion with avoidance.

All associations found in this study were small to moderate. However, because of the rather large number of factors associated with outcomes in this population, a single factor cannot show a large association[75]. The magnitude of our associations are similar to the ones found in other studies, for example Denison found explained variances of 7% for the fear-avoidance variables related to disability[76]. Only a randomized controlled trial targeting patients with an unfavorable profile (mainly patients demonstrating high avoidance) could tell us whether these associations are clinically relevant.

### Limitations

First, the unusual fact that the sample was mainly composed of men may reduce the generalization of the results. Second, due to the real-world clinical setting, we were unable to control for the potentially different therapeutic advice provided by all the members of the healthcare teams about pain, behavior, and physical activity. Indeed, caregivers’ beliefs may also influence patient outcomes[77]. We also cannot exclude that some observations were influenced by factors like physical activity level before rehabilitation. Perceived physical activity before treatment seemed similar in all patterns, but the the leisure subscales of the Baecke questionnaire was the only pertinent available, which may limit the scope of this outcome. Due to the high proportion of non-native French speakers, about 40% of eligible patients had to be excluded because of insufficient fluency in French. This may reduce the generalizability of results, as non-responders often present a different psychosocial profile[61]. The POAM-P and the question about the ability to resume work were not assessed at discharge, thus excluding any possibility to measure changes after treatment. The analyses of co-occurring changes could provide more information on the causal association between behaviors and outcomes. However, our purpose was not to measure the influence of rehabilitation on the activity patterns or on patients’ goals, nor to assess a possible causative effect. Further studies with appropriate designs are needed for that. Finally, as a main limitation of our study, we failed to reconcile contradictory results about patterns already highlighted in previous publications. Nevertheless, we are convinced that our results provide an interesting contribution to this debate. The fact that the same questionnaire (the POAM-P) in a different sample, with different contextual factors, gives different associations between outcomes, pacing, and persistence/overdoing patterns strongly advocates for a more functional and contextual approach like the ones proposed by McCracken, Kindermans and others[9, 17, 22].

### Conclusions

In this study, conducted in a functional and vocational rehabilitation program with patients with chronic musculoskeletal pain, overdoing which appears to be linked to a task-contingent form of persistence[8], was most often related to small or moderate positive biopsychosocial outcomes before and after treatment. Avoidance was related to negative psychosocial outcomes before treatment. The feeling of being able to reach the goal (in our case, resume work after 6 months) was related to a lower avoidance score. Pacing was poorly related to the majority of the measured outcomes. Interpreted in the light of the literature, our results reinforce the idea that activity patterns are probably more context-dependent than inherently functional or dysfunctional. In further studies, it could be interesting to test the efficacy of motivational strategies that may promote functional task-contingent persistence and reduce avoidance of painful tasks, for example, implementation intentions[78, 79].

## Supporting information

S1 File STROBE checklist. (PDF)

S2 File Data file. (XLSX)

S1 Table Associations between the outcomes and the three POAM-P scales. (DOCX)

S2 Table Mean difference between admission and discharge, both unstandardized (delta) and standardized (Cohen’s d). (DOCX)
