# Supplementary material for: Avoidance, pacing, or persistence in multidisciplinary functional rehabilitation for chronic musculoskeletal pain: An observational study with cross-sectional and longitudinal analyses
Source: PLoS One. 2018 Sep 4;13(9):e0203329. doi: 10.1371/journal.pone.0203329 (PMC6122830; doi:10.1371/journal.pone.0203329)
Supplement: S1 Table — (DOCX) [file pone.0203329.s003.docx]

|  | **Avoidance** | | | **Pacing** | | | **Overdoing** | | |
| --- | --- | --- | --- | --- | --- | --- | --- | --- | --- |
| **Outcomes at admission*** | **beta** | **coef** | **p-value** | **beta** | **coef** | **p-value** | **beta** | **coef** | **p-value** |
| **BPI-I** | 0.16 | 0.04 | <0.001 | -0.06 | -0.01 | 0.065 | -0.02 | -0.01 | 0.454 |
| **HADs-D** | 0.17 | 0.09 | <0.001 | -0.10 | -0.05 | 0.004 | -0.14 | -0.07 | <0.001 |
| **SFS/HFS** | -0.09 | -0.53 | 0.012 | 0.01 | 0.06 | 0.743 | 0.17 | 0.98 | <0.001 |
| **6MWT** | 0.02 | 0.35 | 0.590 | -0.10 | -1.54 | 0.006 | 0.10 | 1.53 | 0.005 |
| **PILE** | -0.09 | -0.09 | 0.051 | -0.02 | -0.02 | 0.580 | 0.09 | 0.09 | 0.040 |
| **SRT** | -0.05 | -0.60 | 0.252 | -0.11 | -1.17 | 0.008 | 0.13 | 1.47 | 0.001 |
|  |  | **OR** | **p-value** |  | **OR** | **p-value** |  | **OR** | **p-value** |
| **Ability of Return to Work** |  | 0.97 | 0.035 |  | 1.01 | 0.249 |  | 1.00 | 0.981 |
| **Outcomes at discharge**** | **beta** | **coef** | **p-value** | **beta** | **coef** | **p-value** | **beta** | **coef** | **p-value** |
| **BPI-I reduction** | -0.02 | 0.00 | 0.642 | -0.01 | 0.00 | 0.838 | 0.13 | 0.03 | <0.001 |
| **HADs-D reduction** | -0.07 | -0.02 | 0.087 | 0.03 | 0.01 | 0.416 | 0.10 | 0.03 | 0.007 |
| **SFS/HFS improvement** | -0.05 | -0.18 | 0.275 | 0.04 | 0.15 | 0.293 | 0.12 | 0.48 | 0.001 |
| **6MWT improvement** | -0.05 | -0.54 | 0.280 | 0.03 | 0.32 | 0.459 | 0.12 | 1.27 | 0.003 |
| **PILE improvement** | 0.00 | 0.00 | 0.929 | 0.02 | 0.01 | 0.731 | 0.07 | 0.04 | 0.114 |
| **SRT improvement** | -0.04 | -0.25 | 0.491 | 0.03 | 0.20 | 0.521 | 0.02 | 0.14 | 0.657 |
| ***adjusted for age, gender, pain severity, education level, and trauma location** | | | | | |  |  |  |  |
| ****adjusted for outcome at admission, age, gender, pain severity, education level, trauma location, and length of hospitalization** | | | | | | | | | |

Appendix Table 1 Associations between the outcomes and the three POAM-P scales. Regression models for outcomes at admission are adjusted for age, gender, pain severity and trauma location. Models on outcomes at discharge are further adjusted for the value at admission, and the length of hospitalization. Linear regression models were performed on both standardized (beta) and unstandardized (coef) outcomes.

BPI = Brief Pain Inventory; HAD-D = Hospital Anxiety and Depression Scale - Depression; SFS/HFS = Spinal Function Sort/Hand Function Sort; 6MWT = 6-minute walk test; PILE = Progressive Isoinertial Lifting Evaluation; SRT = Steep Ramp Test.
