# Supplementary material for: Avoidance, pacing, or persistence in multidisciplinary functional rehabilitation for chronic musculoskeletal pain: An observational study with cross-sectional and longitudinal analyses
Source: PLoS One. 2018 Sep 4;13(9):e0203329. doi: 10.1371/journal.pone.0203329 (PMC6122830; doi:10.1371/journal.pone.0203329)
Supplement: S2 Table — (DOCX) [file pone.0203329.s004.docx]

|  | **Total** | | |
| --- | --- | --- | --- |
|  | **delta** | **Cohen's d** | **p-value*** |
| **BPI-I** | 0.69 | 0.37 | <0.001 |
| **HAD-D** | 0.30 | 0.11 | 0.002 |
| **SFS/HFS** | 2.46 | 0.07 | 0.032 |
| **6MWT** | 63.29 | 0.68 | <0.001 |
| **PILE** | 3.16 | 0.61 | <0.001 |
| **SRT** | 31.06 | 0.55 | <0.001 |
| * t-test |  |  |  |

**Appendix, Table 2**

Mean difference between admission and discharge, both unstandardized (delta) and standardized (Cohen’s d).

BPI-I = Brief Pain Inventory - Interference; HAD-D = Hospital Anxiety and Depression Scale - Depression; SFS/HFS = Spinal Function Sort/Hand Function Sort; 6MWT = 6-minute walk test; PILE = Progressive Isoinertial Lifting Evaluation; SRT = Steep Ramp Test.
